# Emerging Technologies for Improving Properties, Shelf Life, and Analysis of Dairy Products

**DOI:** 10.3390/foods13071078

**Published:** 2024-04-01

**Authors:** Golfo Moatsou

**Affiliations:** Laboratory of Dairy Research, Department of Food Science and Human Nutrition, Agricultural University of Athens, Iera Odos 75, 118 55 Athens, Greece; mg@aua.gr

## 1. Introduction

Processing results in several kinds of dairy products with variable properties and shelf lives that preserve and often enhance the unique nutritional and biological value of milk. Apart from nutritional properties, functional and sensory properties are also of great importance for dairy products. There is a constant research effort for the improvement of the production conditions employed in the dairy sector. In this respect, emerging technologies and practices are developed and studied. This research activity has a multidisciplinary character since chemical, physical, microbiological, enzymatic, and microstructural modifications should be considered.

The present Special Issue is a collection of articles related to the application of different types of emerging technologies for the manufacture, analysis, preservation, handling, and analysis of dairy products or dairy components, such as non-thermal processing, enzymatic or fermentation procedures, supplementation with non-dairy substances, and analytical methodologies. Brief information on emerging technologies that have been used in the studies of the present collection, along with the respective articles, is given below.

## 2. Emerging Technologies in the Dairy Sector: Some Research Examples

There are numerous publications in the literature on the application and potential of various categories of emerging technologies in the dairy sector. Some examples are presented below that are related to the articles published in this Special Issue.

One of the most studied topics is the application of emerging non-thermal milk treatments, often in combination with low or moderate heating, as an alternative to prevent the loss of nutritional value and modifications to flavor. The indispensable effect of a treatment alternative to heating should be the elimination of pathogens and other undesirable microorganisms or spores; the requirement for spore destruction depends on the type and shelf life of the final product. In addition, deterioration of flavor and decrease of nutritional value should not be induced or should be at minimum. There are several excellent reviews on this topic. Neocleous et al. [1] and Pegu and Avia [2] reviewed and discussed the effects of high hydrostatic pressure processing (HHP), pulsed electric fields (PEF), ultrasound (US) and hydrodynamic cavitation, UV-C irradiation (200–280 nm), and plasma technology treatments on milk microorganisms, constituents, structural components, and bioactivity. They suggested that HHP and PEF are very effective for the reduction of microbial counts, while ultrasonication and hydrodynamic cavitation under certain conditions can homogenize milk fat and decrease the allergenicity potential of milk. High hydrostatic pressure is one of the most studied non-thermal methods with respect to the inactivation of microorganisms and enzymes and the modifications of structural elements and whey proteins (e.g., [3,4,5,6]). Abrahamsen and Narvhus [7] critically reviewed the literature and concluded that US treatment cannot be considered an alternative to HTST treatment, but the application of US in combination with heating at conditions slightly below typical pasteurization, i.e., thermosonication, has potential interest. They suggest that US can be used to modify the functional properties and the microbial load of dairy products.

Modifications of the structure of milk proteins and consequently of their functionality by cold plasma and the related mechanisms have been reviewed by Sharma and Singh [8]. The effect of cold plasma on sensory and physical properties has been examined in the publication by Nikmaram and Keener [9]. They report that conditions such as gas type, voltage, duration, and plasma source should be optimized in order to combine the excellent lethal effect of this treatment on microorganisms with the preservation of milk quality features. The low installation cost and low energy consumption, the significant reduction in counts of various microbial groups, and the ability to degrade aflatoxins are considered advantages of UV-C irradiation applications in various dairy products. However, the limited penetration capacity of UV irradiation, the appearance of off-flavors, and the tendency of spores to be repaired after UV treatment are considered drawbacks that need further research [10].

The factors that affect the electroporation or electropermealization of the microbial cell membrane induced by PEF treatment of milk and dairy products have been examined in the review by Cavalcanti et al. [11]. In brief, under conditions of PEF treatment that cause irreversible electroporation, the pathogenic and spoilage bacteria, yeasts, and mold counts are efficiently reduced without significant effect on the sensory and nutritional characteristics of milk and various fermented dairy products, but spores exhibit resistance even to severe PEF conditions. Based on the literature, they conclude that PEF technology alone cannot ensure the microbiological quality and shelf life of milk and its products and suggest a combination with another non-thermal approach, such as UV treatment. An interesting application is the use of PEF to cause reversible electroporation that can stimulate the growth and stability of viable microorganisms in yoghurt starters [11]. In general, PEF treatments at low electric strength do not change the structure of proteins. Depending on the conditions and individual proteins, PEF treatment can induce denaturation of whey proteins, while moderate PEF can change the secondary structure of sodium caseinate and unfold the protein molecules [12]. One of the papers in this Special Issue submitted by Sebastià, Calleja-Gómez, Pallarés et al. investigated the potential of the combination of US and PEF treatment to reduce mycotoxin levels. According to the findings, the combination of treatments reduced the levels of Ochratoxin A and Enniatin B up to about half the amount originally inoculated in an orange juice–milk beverage.

The safety and preservation of the quality of milk and dairy products during storage are constantly at the forefront of research and development activities in the dairy sector. As a result, emerging practices, such as various applications of nanotechnology, are investigated. In response to the scientific developments, the European Food Safety Authority (EFSA) has published updated guidance on the risk assessment of nanomaterials to be applied in the food and feed chain, including novel foods, food contact materials, food/feed additives, and pesticides [13]. The most usual application is the incorporation of nanostructures in stable emulsions. There are several techniques and shell materials for the production of nanocapsules, depending on the compound that is encapsulated, the purpose of their use, and the food environment. The functional ingredients of nanocapsules are often bioactive compounds from plant sources or from agro-industrial byproducts that can exhibit antimicrobial, anti-fungal, anti-aflatoxinogenic, or antioxidant activities [14]. Silva et al. [15] discussed the potential of nanoencapsulation of polyphenols in dairy beverages as a means to avoid the destabilizing interactions between phenolic compounds and proteins. Applications of nanotechnology-derived functional edible coatings in yoghurt production have been suggested for the preservation and enhancement of the biological value of yoghurt [16]. A relevant review article is included in the present Special Issue, written by Brandelli, Lopes, and Pinilla. It is an extensive and updated review of the literature for the application of nanostructured antimicrobials in dairy products. Natural antimicrobial compounds, the development of several types of adequate nanostructures—i.e., metal, polymeric, lipid-based, nanofibers, nanofilms, and nanocoatings—and applications in milk and dairy products with a short shelf life, with an emphasis on soft white cheese, are presented. Aspects of potential toxicity are also included.

Other approaches for the extension of the shelf life of dairy products can also be found in the present article collection. The use of bacteriocins as non-chemical bacteriostatic agents in formulations and emerging techniques for their incorporation in the dairy matrix have been recently reviewed [17,18]. This area of research is constantly updated since new bacterial strains often appear in the literature and practices that combine bacteriocins with other antimicrobial agents are developed [19]. The article by Li, Weng, Wu, and Liu in the present collection is a contribution to bacteriocin research. Plantaricin FB-2 from the supernatant of a *Lactiplantibacillus plantarum* FB-2 culture was added to raw and pasteurized milk at different concentrations. They report a significant increase in the shelf life of both milk types at particular levels of addition that coincided with a retardation of the deterioration of sensory attributes during storage.

Spore-forming bacteria are a major concern for the cheese industry, and this is also true for the processed cheeses that are extended shelf-life products. Lysozyme or chemicals are added as spore-inhibition agents. Common chemicals added for this purpose are nitrates, hexamethylene tetramine, and polyphosphates; the latter are used as emulsifying salts, which are normally indispensable additives for this product category [20]. Considering the antimicrobial potential of emulsifying salts, Fusieger, da Silva, Cavicchioli, et al. submitted their study about the effect of two mixtures of polyphosphates/sodium phosphates differentiated in terms of their average chain length and the pH of their solutions. Their experiments were performed in vitro against strains of *B. thuringiensis* and *C. perfringens* and in situ in inoculated processed cheeses manufactured under laboratory or pilot-plant conditions. They confirmed that both treatments reduced *B. thuringiensis* counts while not affecting *C. perfringens*. Based on the literature, they suggest that polyphosphates are sequesters of cations, which are necessary for the enzymatic activity linked to cell division of B. thuringiensis, while higher polyPchain length could be needed for *C. perfringens*.

Strained yoghurt, which is the type of yogurt that results from the partial removal of yoghurt (acid) whey from yogurt and is known worldwide as Greek-style yoghurt, has gained the preference of consumers due to its high protein content, moderate acid flavor, and particular rheological properties. However, many formulations based on mixtures of dairy ingredients rather than milk are labeled as Greek-style yogurts due to their high protein levels [21,22]. Emerging practices or emerging interventions in traditional practices have been applied in the manufacture of this type of yoghurt; there are two examples in this Special Issue. The treatment of yoghurt whey, which is a byproduct, is a matter of great concern mainly due to its high BOD, high mineral content, and low pH, which have a major environmental impact and restrict its potential to be incorporated into other products. There are several practices that partially address the burdens of acid whey, i.e., membrane treatments, biodigesters, neutralization at pH > 6.0, and enzymatic conversion to oligosaccharides [23]. The composition of strained or strained-like yogurts is variable due to the ingredients of the yogurt base, starter cultures, and processing, with emphasis on the method applied for the whey removal [24]. As a result, the respective yoghurt acid whey also has variable composition, especially with respect to protein content [25]. Rocha-Mendosa et al. [23] summarized the uses of yoghurt whey as a part of the formulations of fermented milk beverages, as a cultivation medium for yeast strains or as a medium component for LAB biomass production, as a substrate or substrate component for the production of ethanol, organic acids, hydrogen, amino acids, vitamins, and methane. The contribution of Karastamatis, Zoidou, Moatsou, and Moschopoulou is a study on the combined effect of heat treatment conditions of milk (continuous at 85 °C for 16 s and 100 °C for 16 s or batch at 90 °C for 5 min) and straining conditions (centrifugation at the end of the incubation period or after a 24 h storage period). The results showed that it is possible to decrease the volume of generated acid whey by approximately 10% without jeopardizing the composition and organoleptic acceptance of the product by removing the whey after 24 h of storage, regardless of the conditions of milk heat treatment. Moreover, the higher denaturation degree of whey proteins induced by batch heating increased the gel stability—i.e., higher moisture retention—and reduced the quantity of drained yoghurt whey.

Formulated strained-like yoghurts with high total solids and protein content are produced by means of ultrafiltration or by supplementation of the milk base, usually by casein-based powders, in order to avoid the yoghurt acid whey generated during the straining procedure [22,24,26]. Adequate texture and rheological properties are the main concerns for the research and development of this type of product. Babu, Liu, and Amamcharla, in their contribution to the present Special Issue, suggest the application of air micro- and nanobubbles—using the venturi system or hydrodynamic cavitation—to modify the rheological properties of formulated Greek-style yoghurt with 10% protein. The results showed that the treatment resulted in a more compact microstructure and less viscosity, grain counts, and syneresis compared to the untreated control. Nanobubbles generated by various techniques have emerging applications in the food sector. They are suggested as means to improve processing—because they reduce viscosity—to improve textural properties, intensify sensory characteristics, facilitate freezing, and enhance surface cleaning and defouling [27].

Yoghurt can be the basis for the formulation of many types of dairy desserts by incorporating various categories of substances such as stabilizers, colorants, flavourants, sweeteners, or natural additives of plant origin [28]. Most of them are “yoghurts” with fruit, vegetables, cereals, nuts, seeds, plant/herbal extracts, byproducts, or even waste from fruit or vegetable processing [29,30]. The contribution of Fathy, Abd El-Maksoud, Cheng, and Elshaghabee is a study on the emerging use of byproducts of fruit processing for the improvement of a dairy product. They investigated the potential of the addition of various quantities of sour orange, sweet orange, and lemon peel powders in Acidophilus–Bifidus–Thermophilus (ABT) yoghurt-type products. The addition of up to 0.5% of these powders increased the acidity but did not change the acceptance of the products, whereas it enhanced the antioxidant and antimicrobial potential and the viability of the starter probiotics during storage.

The addition of plants or substances of plant origin, especially spices, to the cheese mass is an old artisanal practice for the extension of shelf life and the enhancement of flavour, which is constantly updated (e.g., [31,32]). In particular, soft spreadable cheese varieties can be an appropriate “substrate” for such interventions due to their texture and high moisture content. The objective of the article by Kondyli, Pappa, Arapoglou et al. was the inclusion of the natural polysaccharide β-glucan extracted from the mushroom Pleurotus ostreatus in the sheep cheese milk for the manufacture of a soft, spreadable cheese (acid and rennet-induced curdling). According to the findings, the addition of β-glucan-rich slurry resulted in a 0.4% β-glucan content in cheese and increased the cheese moisture from 72.6 to 74.5% without affecting the pH, colour, viscosity, proteolysis, lipolysis, or antioxidant potential. Interestingly, the supplementation increased the flavour score of cheese within 2 to 3 weeks of storage. These results indicate that it is possible to exploit the important biofunctionality of this compound by manufacturing this type of product. The use of different types of β-glucans from various sources (e.g., oats, barley, mushrooms, and yeast) in food formulations has been initially investigated due to their diverse biofunctionalities, such as antioxidant properties, positive effects on blood sugar and cholesterol levels, immunomodulating potential, antitumor effects, and protection from the effects of stress [33]. In addition, β-glucans exhibit important technological properties, i.e., binding of free moisture, mimicking milk fat, formation of structure, increase of cheese yield, and increase of the ice cream overrun. The interesting results of relevant studies—although some of them are contradictory—have been recently reviewed [33,34,35].

It is well established that a variety of peptides that exhibit various types of biofunctionality are encoded in the sequences of milk proteins. These peptides can be liberated during gastrointestinal digestion, by fermentation, or by enzymatic hydrolysis. The latter has been successfully applied to the production of food supplements, nutraceuticals, and infant formulas and has been an evolving research topic in the dairy field. Of specific interest is the development of whey protein hydrolysates (WPH), which is a constantly updated technology with commercial and research applications. WPHs exhibit modified functionality and eventually improved biofunctionality compared to the initial whey protein substrate [36,37]. The outcome of hydrolysis depends on many factors, such as enzymes, physicochemical conditions, substrate pretreatment, and incubation conditions [38,39]. The objective of the article submitted by Sakkas, Lekaki, and Moatsou was to produce tryptic hydrolysates of two whey protein concentrates of sheep/goat origin with a high residual intact protein content without pH control to avoid dilution and an increase in the ash content. The marginal conditions for trypsin activity applied in the experiments resulted in a limited degree of hydrolysis, which coincided with a dramatic increase in ACE inhibitory activity without any decrease in radical scavenging or chelating activity. The emerging approach of this study, i.e., short reaction time, mild heat treatment applied for the inactivation of the enzyme, and the absence of additives, proved to be an appropriate combination for the preparation of hydrolysates that remained stable under cold storage and contained a considerable amount of intact native whey proteins.

Due to the complexity of the matrix of milk and dairy products and the demands of legislation with respect to their manufacture, storage, and marketing, all categories of analytical methods are applied—often in combinations—in dairy research, product development, and quality control (e.g., [40,41,42,43,44]). The study of Zobkova, Yurova, Semipyatniy, et al. proposes an innovative application of a biotesting procedure based on the assessment of protozoa metabolism. The research is a continuation of a previous study by the same group [45] for the development of a test for fermented milk called relative biological value (RBV) using *Tetrahymena puriformis* as a test organism. By applying this type of biotest, they could optimize the formulation with respect to the biofunctional and rheological properties of the final product. In the present article, they studied the RBV evaluation procedure in acid cheese curd manufacture. The parameters that proved to be the most significant were the heating conditions of the milk and the scalding temperature of the curd pieces. Statistical analysis showed the optimal manufacturing conditions for this type of curd that maximized the RBV. A second paper in the present Special Issue that has an objective in the field of milk and dairy analysis has been submitted by Fan, Li, Zhang, et al. They present the design and application of a qPCR method for the qualitative and quantitative determination of strains of six lactic acid bacteria in fermented milk in comparison to the plate count method and flow cytometry. They suggested *tuf* as a target gene instead of the usually utilized 16S rRNA gene and concluded that the results obtained by their novel approach were more reliable than those obtained by the other two methods.

## 3. Conclusions

There are many innovative suggestions and emerging practices for the treatment of milk and the manufacture of dairy products that focus on the enhancement of safety, functional and nutritional properties, and the reduction of energy consumption and environmental impact. However, milk is a very complex biological material, and its constituents are in a state of rather fragile dynamic equilibrium. Moreover, the properties and shelf life of dairy products are highly variable. Any treatment that does not “respect” these facts destabilizes milk and induces defects in the dairy products. An emerging method, in order to become a unit operation that replaces a traditional one, has to ensure the safety, shelf life, and nutritional value of dairy products, be continuous, have a high capacity, and have reasonable installation time and installation/maintenance costs. Therefore, extensive, specific, multidisciplinary, and time-consuming experiments are needed before the application of an emerging technology or practice in the dairy sector. The collection of articles published in this Special Issue can be considered contributions to this very demanding research field.

## Data Availability

No new data was created or analyzed in this Editorial.
